# Supplementary material for: DMRforPairs: identifying Differentially Methylated Regions between unique samples using array based methylation profiles
Source: BMC Bioinformatics. 2014 May 15;15:141. doi: 10.1186/1471-2105-15-141 (PMC4046028; doi:10.1186/1471-2105-15-141)
Supplement: Additional file 2 — DMRforPairs output for the comparison of A431-MCF7 and NA17018-NA17105. Please start from the HTML files in each folder. Available via the BMC Bioinformatics website. [file 1471-2105-15-141-S2.zip › 1394847754114233_MOESM2_ESM/A431_MCF7/figures/28153.pdf]

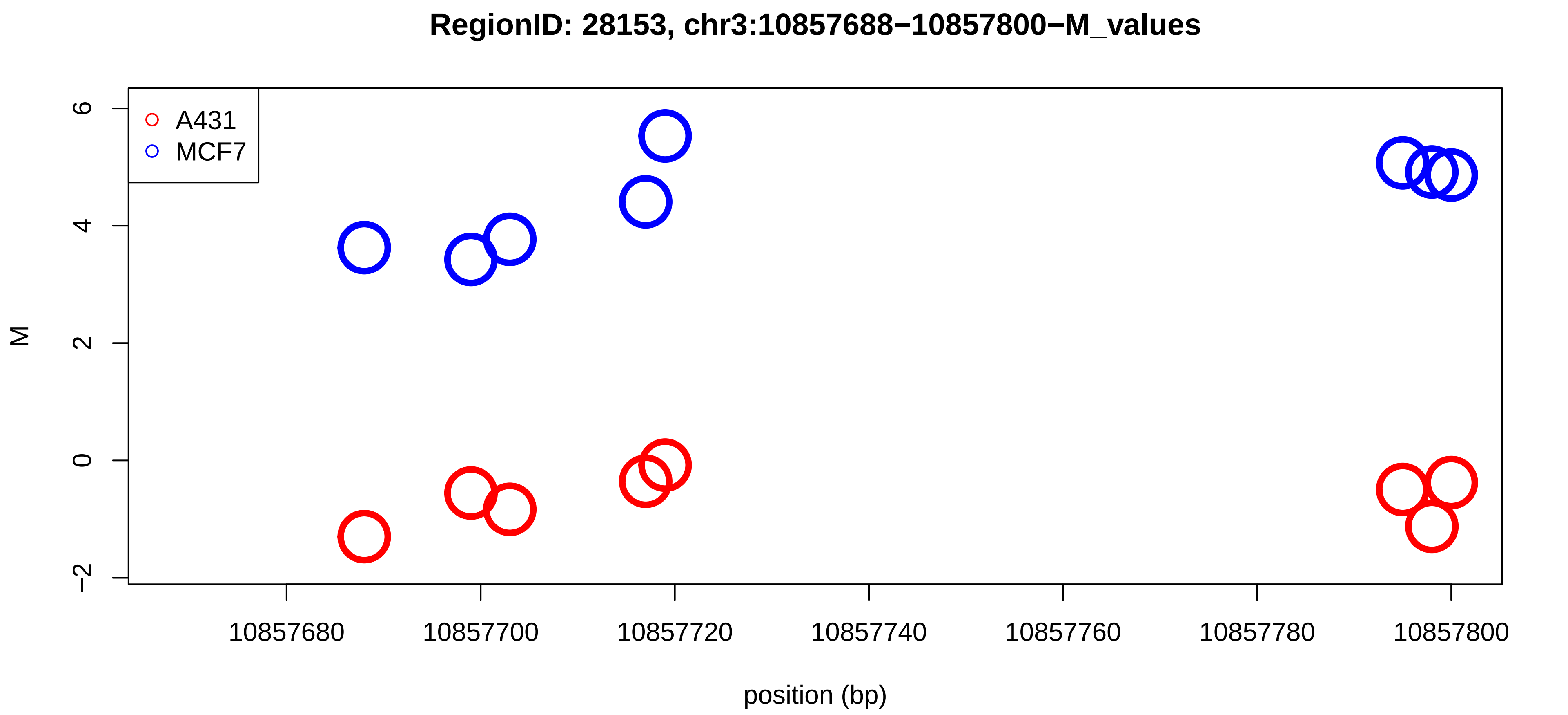
RegionID: 28153, chr3:10857688−10857800−M_values
A431
MCF7
M
−2
0
2
4
6
10857680
10857700
10857720
10857740
10857760
10857780
10857800
position (bp)

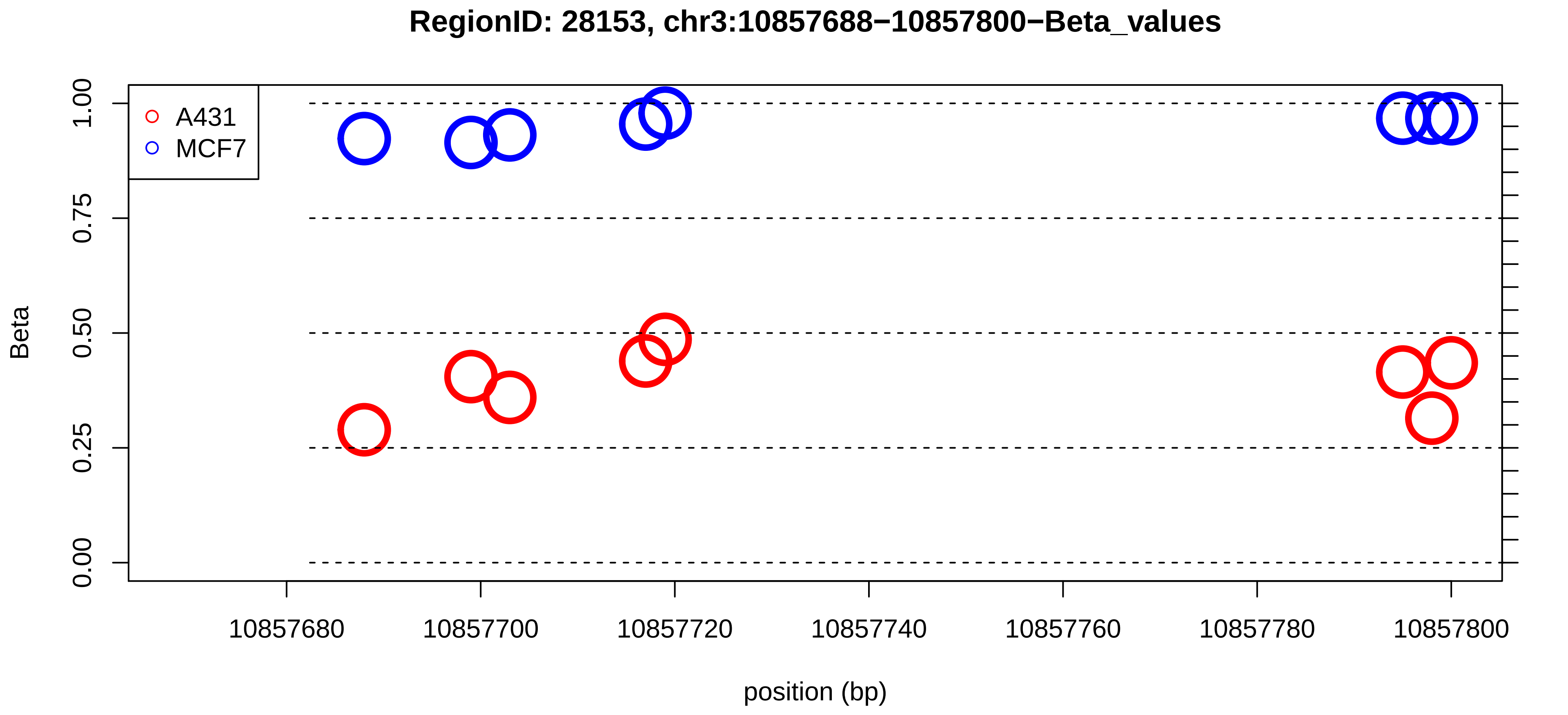
RegionID: 28153, chr3:10857688−10857800−Beta_values
A431
MCF7
Beta
0.00
0.25
0.50
0.75
1.00
10857680
10857700
10857720
10857740
10857760
10857780
10857800
position (bp)

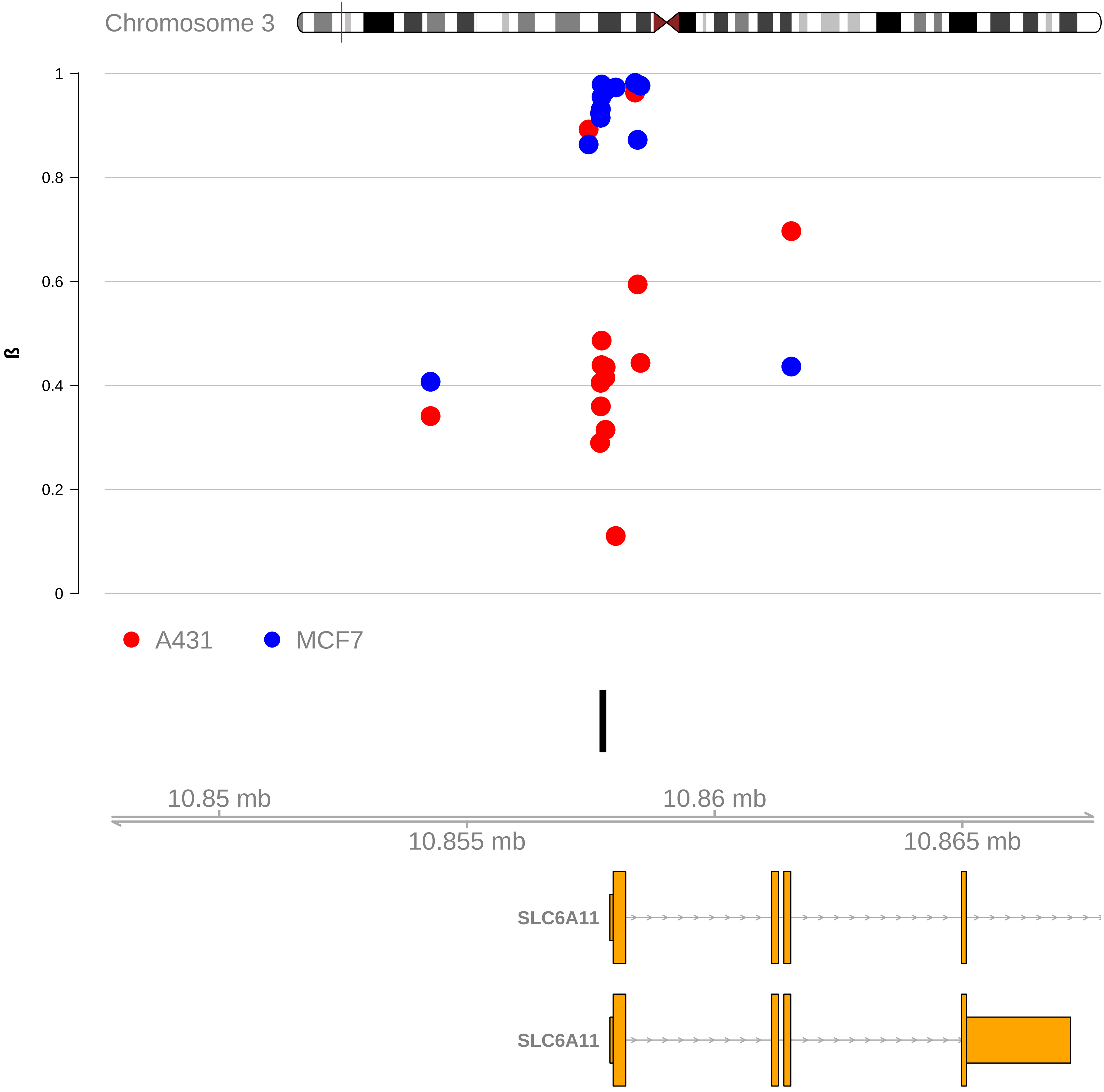
Chromosome 3
1
0.8
0.6
0.4
0.2
0
ß
A431
MCF7
10.85 mb
10.86 mb
10.855 mb
10.865 mb
SLC6A11
SLC6A11
